# Bibliographical Notices

**Published:** 1849-01

**Authors:** 


### BIBLIOGRAPHICAL NOTICES.

*An Introductory Lecture on the Coinciding Tendencies of Medicines.* By JARED P. KIRTLAND, M. D., Prof. of Theory and Practice of Medicine, and Physical Diagnosis, in the Medical Department of the Western Reserve College, at Cleveland, Ohio.

By the expression, "Coinciding Tendencies of Medicines," (which is taken from the Treatise of Miner and Tully, on Fever, published some twenty-five years ago,) the writer appears to mean, simply, that medicines, if injudiciously prescribed, may exert an unfavorable, instead of a favorable influence. The expression is not strictly correct, inasmuch as it is plain that remedies which are injudiciously prescribed, do not, in all instances, exert tendencies similar to the tendencies of the diseases for which they are prescribed; that is to say, they may not do injury by their coinciding tendencies, but often by modes of action quite different from the morbid actions to which they are added.

The principle which the subject of the discourse involves, is a truism certainly having no claims to originality or novelty. Yet the author seriously avers that it is passed over by medical writers, and mentions Miner and Tully's Treatise, as the only work in which it is duly considered.

It is with still greater surprise that we find it stated in the discourse, that the Cleveland Medical School is the only School in the country in which the subject receives attention even for a single hour.

If the Professor really intends to propound the theory that the hurtful results of medicines are, in their nature, *coinciding*, then we admit that the honor of the doctrine is to be divided between Miner and Tully, the Cleveland medical school, and himself. The idea, however, that he intends so to be understood, seems too absurd to be entertained. If he means only that the misnomer, "coinciding tendencies," is peculiar to the trio just mentioned, this would be readily enough conceded; but it would be too frivolous to claim any exclusive merit on that score. Either of these interpretations of the author's reasoning is probably incorrect; and, yet, it is hardly less inconceivable that he should suppose the injurious influences of medicines to be a subject overlooked by medical writers, and that it does not receive at other institutions than Cleveland more consideration than could conveniently be compressed into a single hour! We can see no alternative but to understand the language of the writer in this sense, strange as it may seem, coming, as it does, from one presumed to be conversant with the medical literature of the day. We do not deem it in the least necessary to argue against such an extraordinary position.

The following quotation will, we think, strike our readers with as much surprise as it did us:

"Acute disease always occurs as one of two qualities—either *sthenic* or *asthenic*. On employing these terms, permit me to say, that we use them only in their usual acceptation, and not with any reference to the Brunonian system. * * The *sthenic* is attended with a morbid accumulation of vitality, the principle of life, which imparts an increased power of acting. The *asthenic* is, on the other hand, attended with a diminished amount of vitality; consequently the power to act, in the system, is less than during health."

Prof. K. disclaims any reference to Brunonism in the use of the Brunonian terms contained in the above quotation; but how can the doctrine be considered other than the Brunonian doctrine? In his definition of the terms, they have precisely the signification which belonged to them as employed by Brown and his followers, to wit: denoting a *plus* and *minus* quantity of a supposititious principle called vitality. The terms are rarely used by late medical writers, and when used, they are generally understood to have a figurative sense. Their usual acceptation is certainly different from the sense which the writer of this discourse attaches to them. The expression *vital principle* is also employed in a metaphorical sense for the sake of convenience, but the idea of an entity, under this, or any other title, presiding with a sort of instinctive intelligence over the functions of the organism, we had thought was well nigh obsolete, having been laid on the shelf with other by-gone speculations. The existence of such an entity, in the foregoing quotations, is plainly assumed; imparting increased power in *sthenic*, and experiencing diminution of its natural power in *asthenic* diseases. We opine that our readers would not thank us for undertaking to disprove the correctness of the Brunonian theory.

The following concluding paragraphs contain assertions upon which we have already commented, together with some points to which we shall devote a remark or two:—

"To the young gentlemen, who are about to attend the course of instruction in these Halls, I would, in a particular manner, address the subject of the coinciding tendencies of medicine.

Important as it is, this Institution, I believe, is the only one in our country, in which an hour is devoted, during each term, to its consideration. It is slightly alluded to in a few authors. In Miner and Tully's work on Fevers, it occupies an interesting chapter.

With those gentlemen I was intimately associated in early life, and to them was originally indebted for many of the views I have laid before you on this occasion. An extensive experience has subsequently confirmed their correctness.

It is my ardent wish that they may prove to you, in your future practice, as valuable guides as they have to me in days that are past.

From the moment the student of medicine first opens a treatise on Theory and Practice, till he receives the honors of the Institution, he is taught, in most schools, to consider medicines as simply the antagonists of disease; and is not initiated into the important secret, that medicines, under certain circumstances, may themselves become the source of disease. He enters upon the stage of action with the firm persuasion, that he has only to administer medicine with a bold and liberal hand, and he will at once convert disease into health; that, like Alladin in oriental fable, he has only to rub the lamp, command, and it will be executed. Experience soon convinces him that his views are incorrect. On treating disease, he finds himself surrounded with new and anomalous symptoms, of which he had no previous conception, and which increase by every effort at extirpating them —ignorant of the source of the perplexities—he becomes distrustful of the certainties of medical science.

If he be a man of principle, he will most likely retire in disgust from the profession, and ever after remain the most confirmed of medical skeptics. If, by chance, he is destitute of principle, he will probably continue to practice it, as a mere trade or art, for the purpose of obtaining his daily bread, but will dwindle into insignificance as he advances in age.

You have been furnished with a key to such perplexities.

These doctrines, I am aware, are not palatable to young and ardent students. They dislike to be told that prudence, and not boldness, should be exercised in treating the sick.

These principles I have taught many years. They have been received with coldness by a majority of my classes. That matters not; it is not my duty to pamper students' tastes in my teachings; but it is both my duty and pleasure to fit them to be skillful and judicious practitioners.

I know that in a few years you will recall to mind these sober truths, and put upon them a just estimate. Receive them, then, young gentlemen! store them away in your memories; and when you have matured in experience and professional knowledge, recall them to mind, and test them in the strictest manner."

An analysis of the above, develops the following declarations viz: (*a*) The principles set forth in this discourse are not taught at most institutions; but at Cleveland an hour's attention is devoted to them, although received with coldness by the majority of the classes. (*b*) Students educated at other institutions where these principles are not inculcated, after entering upon practice, become distrustful of the certainties of medical science; and, if they are men of principle, they retire from the profession in disgust, remaining the most confirmed of medical skeptics; but if, by chance, they are destitute of principle,they will probably continue to practice the profession as a mere trade.

The logical deduction from the two postulates just given, is, that all practitioners of medicine who have not enjoyed the advantages of the information communicated in the aforesaid hour at the Cleveland Medical

College, are devoid of principle, and practice their profession as a mere trade!

With such views, the publication of this address is certainly evidence of the disinterestedness and philanthropy of the author; for, by its perusal, possibly some of the few men of principle who enter the profession unacquainted with that necessity of going to Cleveland which has heretofore existed, may be prevented from leaving in disgust. At the same time, the classes annually assembling at Cleveland will be better satisfied, inasmuch as the author intimates that the hour devoted to this discourse has been by no means acceptable to them.

---

A *Treatise on Etherization in Child-birth. Illustrated by five hundred and eighty-one cases.* By WALTER CHANNING, M. D., Professor of Midwifery and Medical Jurisprudence in the University at Cambridge. Boston: William D. Ticknor & Company, 1848. 8 vo. pp. 400.

This volume will commend itself to the admirers of elegance in typography. It is beautifully printed with large clear type, on fine paper, and, indeed, is creditable even to Boston publishers. We speak of its external recommendations first, simply because, with the book before us, they first suggest themselves. Its attractiveness, by no means, chiefly lies in externals. If we are not mistaken, it will prove as acceptable, as it is opportune. The importance of the subject is obvious. It is a subject with regard to which a large proportion of the profession are waiting for facts sufficiently numerous and authenticated, to determine their opinions and practice. Such a collection of cases, emanating from a quarter which secures confidence in their validity, cannot but command attention and interest. Prof. Channing remarks upon the appropriateness of a work upon that subject being issued at the place where he resides. Without concurring fully in the unqualified attribution of the honor of the discovery of anasthetic inhalations to Boston, it will not be denied that the importance of the discovery here first became appreciated sufficiently for its practical availableness to be fully tested, and, by the experiments repeated and extended at the Massachusetts General Hospital, subsequent observations in all countries may be said to have been originated. We would respectfully remind our Boston brethren not to overlook the fact that successful experiments for the same end were made prior to those of Jackson and Morton, by a resident of Hartford, Connecticut. We would say, in the words of Comdore Perry after the battle of Lake Erie, "there is glory enough for all." Boston has acquired honor enough in this matter, to be able to afford all that in justice, or generosity, is demanded on behalf of the memory of Dr. Wells. We agree, then, with our distinguished friend, that such a work comes with singular propriety from the Boston press; and we would add, what the author would be the last to intimate, that such a work could not, with so much propriety, come from any other pen. Professor Channing was the first in this country to enter upon a series of experimental observations of the applicability of anæsthetic agents to midwifery. He is thus identified with the early history of the subject, and it is fitting that he should take the lead in its bibliography. In addition to this, we know of no one whose position as teacher of obstetrical medicine, and whose character as a medical philospher and writer, should better entitle him to such precedence.

We have said that, as regards the application of anœsthetic agents to midwifery, a great portion of the profession are, as yet, undecided. There exists a suspension of judgment, not only from that cautiousness which is reasonable and proper, but because eminent teachers of midwifery differ on the question. With regard to this difference, however, it is worthy of note, that the advocates of the practice base their opinion on the facts disclosed by their experience, while the opponents (so far as we know) have come to a decision from speculative reasoning, mainly, of the merits of the subject. Our own opinion would possess very little importance, nor do we feel called upon to express an opinion. In fact we have none. We have not yet made trial of etherization in obstetrical practice. We shall peruse Prof. Channing's treatise with care and interest, so soon as our engagements will permit. In the meantime, we would urge our readers, most of whom have the advantage of us in leisure, to make the same resolve, and act upon it. In examing the work, we find it is chiefly made up facts, in other words, cases occurring in the practice of the author, and of his numerous friends. This is the kind of book which is wanted. And having stated this, we have not the vanity to suppose that it would add to our recommendation of the work, if we were prepared to express opinions formed after the careful perusal which we are impatient to bestow upon it. Let the reader obtain the book, study the facts, and draw his own conclusions.

---

*Analytical Compendium of the various branches of Medical Science, for the use and examination of Students.* By John Neill, M. D., etc. and Francis Gurney Smith, M. D., etc. Phildelphia: Lea & Blanchard, 1848. 12 mo.

The usefulness of works of this description has been called in question. We conceive that they may be useful to the student, if properly used; that is, their objects are legitimate, but they are capable of being applied to wrong purposes. If they were to be made subservient to what has been significantly called a *cramming* process, by which a superficial, smattering knowledge of the several branches of medical science, only, is obtained, they would be, beyond a doubt, not merely among the *excrescences,* but among the *nuisances* of medical literature. But when, on the other hand, they are made subsidiary to other works, and to oral teaching, assisting to review the knowledge acquired through the latter channels, they are both convenient, and useful. They may also be of assistance to the the practitioner by affording an easy method of reviewing his knowledge, and refreshing his recollection of the more important facts of medical science. As we do not deem the liability to abuse, a valid objection to the proper use of any good thing, we are not opposed to the publication of such works, and the above seems to be prepared with care and judgment. It is divided into seven parts, to wit, Anatomy, Physiology, Surgery, Obstetrics, Materia Medica, and Therapeutics, Chemistry, and the Practice of Medicine.

---

*The Principles and Practice of Modern Surgery.* By ROBERT DRUITT, Fellow of the Royal College of Surgeons. A new American, from the last and improved London edition. Edited by F. W. SARGENT, M. D., author of Minor Surgery, etc. Illustrated with one hundred and ninety-three engravings. Philadelphia: Lea & Blanchard. 1848. 8 vo. pp. 576.

As this work has been heretofore ably reviewed in this journal by a correspondent and colleague, it is only necessary to chronicle the fact of a new edition, which the author informs us, in the preface, has been considerably enlarged by the addition of new practical matter, furnished by surgical improvements that have been made since the first edition was published. The previous American editions were edited by Dr. Joshua B. Flint, of Louisville, Ky. Owing to the distance of Dr. Flint from Philadelphia, and the difficulty of superintending its progress through the press, he declined further editorial connection with the work, and his place has been filled by Dr. Sargent, favorably known by his recent Treatise on Minor Surgery. Dr. Sargent has retained the notes to former editions by Dr. Flint, excepting those incorporated by the author in the text, and has added such "results of the skill and industry of American Surgeons as appeared most worthy of mention."

*Harrison's Anatomy; a Text Book of Practical Anatomy.* By Robert Harrison, M. D., M, R. I. A; Professor of Anatomy and Surgery in the University of Dublin, with Additions by an American Physician. New York. S. S. & W. Wood; No. 261 Pearl St. 1848.

The well merited reputation of the Dublin Dissector by the same author, led us to anticipate a valuable addition to this department of science. Examination fully confirms our expectations.

The work before us is a new edition of the Dublin Dissector, much improved. Enlarged to the extent of 720 octavo pages, it appears to contain all that is necessary for the objects set forth in the preface, viz., to direct the student in the manner best adapted to faciliate his enquiries, and to perfect his knowledge of anatomy.

The arrangement of the work is such as to be readily consulted in the Dissecting Room, for which it is especially designed.

The additions by the American Editor, though not numerous, will be gladly received. Sufficiently minute, without tedious prolixity, and abounding in suggestions of practical value, it cannot fail to be a favorite work with the student of anatomy. Its utility is very much increased by the introduction of numerous plates, and the addition of a copious index.

It is a valuable work, and we cheerfully recommend it. C. L. F.

---

*Meachem on Dysentery.*—A practical paper in your last number was this,—replete with good sense when its author reflects, and utters himself. The Ontario Medical Society did well in advising its publication;—would it not have been better to have noted and given heed to this statement of other's treatment, p. 409;—an inadvertence so frequently met with, we are now induced to speak to it:—"Some *relied* mainly on astringents, such as acetate of lead combined with Dover's powder." A *reliance* probably suggested by first thought facility of Opium and Ipecac mixed, and at hand, in Dover's powder. We opine an insoluble sulphate of lead, a product of this combination, would not contribute materially as a curative in any disease, and, as a *reliable* astringent base, would be decidedly "expectant" for a result, in an epidemic dysentery, which, under the best treatment, in numbers attacked, severity, and fatality, is believed the past season, to have out-Heroded itself. W. T.

---

*Gardner's Medical Chemistry for the Use of Students and the Profession.* Philadelphia: Lea & Blanchard. 1848. 12mo. pp. 396.

A treatise of chemistry adapted specially to the wants of medical students, is certainly one of the desideratas, or, to speak technically, one of the

"indications" of the times; and as the first effort to "fulfil" this, we hail the appearance of the present volume. We have not yet been able to give it more than a hasty glance; but its style appears to be clear and lucid, and the arrangement of its subjects judicious and well proportioned. In one respect it commends itself to our eyes before opening its leaves, and that is in the moderate distance between its covers. If the book, whatever its subject, is to be a text book, give us a thin one. And this is a duodecimo of less than 400 pages. G. H.

---

*Medical Lexicon.* A Dictionary of Medical Science; containing a concise explanation of the various subjects and terms; with the French and other synonymes; notices of climate, and of celebrated mineral waters; formulæ for various officinal and empirical preparations, etc. By Robley Dunglison, M. D., Prof. of the Institutes of Medicine, etc., in Jefferson Medical College, Philadelphia. Seventh edition. Carefully revised and greatly enlarged. Philadelphia. Lea & Blanchard. 1848. 8vo.

This work has passed through six previous editions, each of which has embraced copious additions, and other improvements. The author states that in the present edition he has added between *six and seven thousand terms.* The size of the page has been increased, and the work enlarged by more than one hundred pages. It is doubtless now the most comprehensive and best English dictionary of medical terms extant.

---

*Essays on Infant Therapeutics; to which are added Observations on Ergot, and an Account of the Origin of the Use of Mercury in Inflammatory Complaints.* By John B. Beck, M. D., Prof. of Materia Medica in the College of Physicians and Surgeons of the Univ. of the State of New York, etc, etc., etc. New York. W. E. Dean, Printer and Publisher, No. 2 Ann Street, 1849.

The essays upon infant therapeutics, are the communications on the effects of opium, emetics, blisters and mercurials, on the young subject, which have appeared in different medical journals within the last two years, and have been presented in the eclectic department of previous numbers of this Journal. Collected together, with articles on ergot, and the use of mercury, which had previously been communicated, they make a thin duodecimo of a little over one hundred pages, and will doubtless be acceptable, in this form, to the profession.

---

*An Introductory Lecture to the Medical Class of the University of Louisville.* By Lunsford P. Yandell, M. D., Professor of Chemistry and Pharmacy.

This is a sensible discourse, written in a chaste dignified style. The reader rises from its perusal with a high regard for the author's spirit and ability.

*The Universal Guide to Health, by a rational Course of Food and Diet.* By ANDREW COMBE, M. D. Seventh Edition. Buffalo. G. H. Derby & Co. 1849. 12mo. pp. 310.

This well known and most admirable work of Dr. Combe, is now very justly regarded as standing at the head of all publications relating to the subject of which it treats. The author was one of the first to perceive the real importance of physiological knowledge to the general public, as he has been altogether the most successful in his mode of conveying it. The amount of good which his works have been instrumental in doing, both in Europe and this country, is incalculable; and they are destined to exert a still wider influence than they yet have done. The present Essay relates chiefly to the function of digestion and the principles of dietetics, and to say that it is the best work on the subject, both for the professional reader and the public, is but saying what it universally known and acknowledged. —For sale at Derby's. C. A. L.

---

*Transactions of the American Medical Association.*—Inquiries are frequently made respecting the mode of procuring copies of the above. The following information on the subject is copied from the Medical News:—

"Medical Societies which have been represented in the Association will be furnished copies on the same terms as members, (viz: three copies for five dollars,) on remitting the amount to the Treasurer.

To other persons the Transactions will be furnished at the rate of two dollars per copy in paper covers, done up for mail, or two dollars and fifty cents in embossed cloth, on remitting the amount direct to Messrs. Lea & Blanchard, Philadelphia. Or orders left with booksellers will be executed by Messrs. Lea & Blanchard.

---

*Valedictory Address to the Students of the Medical College at La Porte, Indiana.* By A. B. SHIPMAN, M. D.

We should have acknowledged the receipt of a copy of this discourse long ago. It was delivered to the graduates at the close of the last session, and contains judicious counsel with reference to professional conduct and success.

